# Air pollution before and during the COVID-19 pandemic: changes in risk of acute myocardial infarction

**DOI:** 10.1093/eurheartj/ehag102

**Published:** 2026-02-13

**Authors:** Masanobu Ishii, Taishi Nakamura, So Ikebe, Yasuhiro Otsuka, Kenichi Tsujita

**Affiliations:** Department of Medical Information Science, Graduate School of Medical Sciences, Kumamoto University, 1-1-1, Honjo, Chuo-ku, Kumamoto 860-8556, Japan; Department of Cardiovascular Medicine, Graduate School of Medical Sciences, Kumamoto University, 1-1-1, Honjo, Chuo-ku, Kumamoto 860-8556, Japan; Department of Medical Information Science, Graduate School of Medical Sciences, Kumamoto University, 1-1-1, Honjo, Chuo-ku, Kumamoto 860-8556, Japan; Department of Cardiovascular Medicine, Graduate School of Medical Sciences, Kumamoto University, 1-1-1, Honjo, Chuo-ku, Kumamoto 860-8556, Japan; Department of Cardiovascular Medicine, Graduate School of Medical Sciences, Kumamoto University, 1-1-1, Honjo, Chuo-ku, Kumamoto 860-8556, Japan; Department of Cardiovascular Medicine, Graduate School of Medical Sciences, Kumamoto University, 1-1-1, Honjo, Chuo-ku, Kumamoto 860-8556, Japan

**Keywords:** Air pollution, Acute myocardial infarction, MINOCA, PM2.5, Prevention

## Abstract

**Background and Aims:**

Ambient PM2.5 exposure is a known risk factor for acute myocardial infarction (AMI). Myocardial infarction with non-obstructive coronary arteries (MINOCA) represents a distinct subtype of AMI from myocardial infarction with obstructive coronary disease (MI-CAD). Public health measures during the COVID-19 pandemic may have altered exposure patterns, offering a natural experiment. This study investigated changes in the association between short-term exposure to PM2.5 and AMI subtypes following the COVID-19 pandemic.

**Methods:**

Using data from the nationwide JROAD-DPC database, this study analysed 270 091 first-admission AMI patients, including 23 037 MINOCA, between April 2012 and March 2022. A time-stratified case-crossover design with conditional logistic regression estimated the odds ratio (OR) for each 10-μg/m³ increase in PM2.5 exposure at a 2-day lag. The study period was divided into pre-pandemic and post-pandemic phases using 7 April 2020, as the threshold. Sensitivity analyses used alternative pandemic onset dates.

**Results:**

Each 10-μg/m³ increase in lag-2 PM2.5 was significantly associated with an increased risk of AMI. Although the ORs for overall AMI and MI-CAD remained largely unchanged between periods, a significant attenuation of the PM2.5-related risk for MINOCA was observed (pre-pandemic OR 1.303, 95% confidence interval [CI] 1.005–1.688; post-pandemic OR 1.230, 95% CI: .973–1.555; *P* for interaction = 0.017). Sensitivity analyses confirmed these findings.

**Conclusions:**

Short-term exposure to PM2.5 is associated with increased risk of AMI. The observed reduction in MINOCA risk following the COVID-19 pandemic suggested that public health interventions aimed at reducing air pollution exposure may reduce cardiovascular risks, particularly in specific AMI subtypes.

## Structured Graphical Abstract

**Structured Graphical Abstract:**
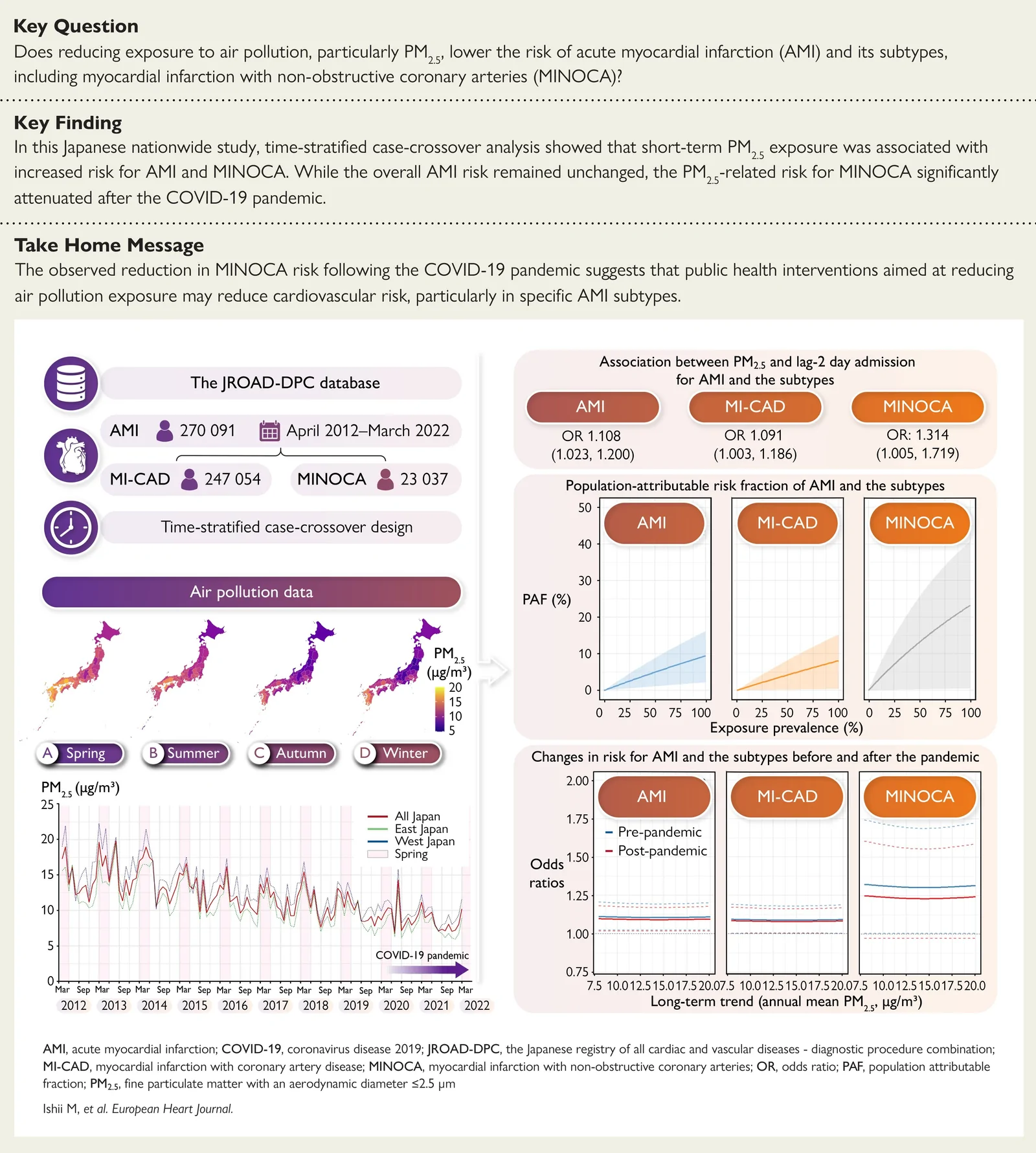

**See the editorial comment for this article ‘A natural experiment in the air: particulate matter and acute myocardial infarction subtypes during the COVID-19 pandemic', by G. Guida *et al*., https://doi.org/10.1093/eurheartj/ehag218.**

## Introduction

Acute myocardial infarction (AMI) remains a leading cause of morbidity and mortality worldwide,^1^ while myocardial infarction with nonobstructive coronary arteries (MINOCA) represents a subset of AMI characterized by heterogeneous mechanisms and clinical outcomes.^2–5^ The global burden of disease attributable to ambient air pollution, particularly fine particulate matter with an aerodynamic diameter ≤2.5 µm (PM_2.5_), is substantial, with established risk for cardiovascular morbidity and mortality.^6–8^ Previous animal and epidemiological studies suggested that PM_2.5_ exposure triggers systemic inflammation, oxidative stress, and endothelial dysfunction, which are critical pathways in atherosclerosis and microvascular dysfunction.^8–11^

Previous investigations have demonstrated seasonal and regional variability in PM_2.5_-associated cardiovascular risks.^12,13^ Elevated PM_2.5_ concentration in spring was associated with higher rates of AMI, including MINOCA, potentially due to transboundary pollution and specific seasonal sources.^12,13^ However, few studies have assessed how these risks differ for MINOCA vs obstructive AMI, particularly in the context of the COVID-19 pandemic. Measures implemented during the pandemic, including mask-wearing and mobility restrictions, might have altered air quality patterns globally and may have contributed to reduced individual exposure to air pollution. These changes provide an opportunity to evaluate whether such interventions could reduce PM_2.5_-related risks.

Based on these observations, we hypothesized that the pandemic altered exposure–outcome relationships due to changes in air pollution exposure patterns and that public health measures may have contributed to a reduced risk of PM_2.5_-related AMI. The aim of this study was to estimate whether avoiding short-term exposure to PM_2.5_ could reduce the risk of disease development through a natural experimental study utilizing nationwide data from the Japanese Of All cardiac and vascular Disease-Diagnostic Procedure Combination (JROAD-DPC) database.

## Methods

### Study participants

The data of patients admitted for AMI at the Japanese Circulation Society (JCS)-certificated hospitals between 1 April 2012 through 31 March 2022 were obtained from the nationwide administrative Diagnostic Procedure Combination (DPC) database, the JROAD-DPC database, as previously described.^14–16^ The details of study enrolment, exclusion criteria, and the classification algorithm for AMI subtypes have been described elsewhere.^12,13^ Briefly, patients admitted with a diagnosis of AMI (*n* = 480 806) were identified by using the International Classification of Diseases, Tenth Revision (ICD-10) codes for AMI. Patients who underwent re-hospitalization for AMI (*n* = 8638) and had an existing myocardial infarction (*n* = 11 515) were excluded, as recurrent AMI is likely attributable to factors other than air pollutant exposure. Patients who neither underwent coronary angiography nor revascularization (*n* = 57 370) and those not admitted as emergency cases (*n* = 7228) were also excluded. After exclusions, 396 055 AMI patients admitted for the first time during the study period were identified. Additionally, patients with troponin-positive non-obstructive coronary arteries due to non-coronary causes (*n* = 4254) and those with missing data on air pollutants and environmental factors (*n* = 121 710) were excluded. Finally, 270 091 patients were analysed (see Supplementary data online, *Figure S1*). These patients were further divided into two subgroups: Myocardial infarction with coronary artery disease (MI-CAD) and MINOCA, based on a validated algorithm combined with ICD-10, diagnostic, and procedure codes.^12^ A previous validation study demonstrated that the MINOCA algorithm has a specificity and positive predictive value of 100%. However, its sensitivity was limited, with 27.0% for identifying working diagnoses of MINOCA and 45.5% for identifying true MINOCA cases.^12^

This study was conducted in accordance with the Declaration of Helsinki and its amendments and was approved by the ethics committees at Kumamoto University (Rinri-No. 2724). The requirement for individual informed consent was waived due to anonymization of all patient data.

### Study design

This observational study leveraged a natural experiment design.^17,18^ In Japan, the government declared a state of emergency on 7 April 2020, which we used as the exogenous threshold to divide the study period into pre-pandemic and post-pandemic phases. This natural segmentation enabled the assessment of variations in short-term exposure to PM_2.5_ and their association with AMI risk. A time-stratified case-crossover design^19,20^ and conditional logistic regression model estimated odds ratios (OR) for PM_2.5_-related risk, while assessing potential interactions between exposure and the pandemic period. This quasi-experimental framework strengthens causal inference from observational data by utilizing an external, unmanipulated intervention.^17,18^

This case-crossover design assigned ‘case’ and ‘control’ days within the same patient to adjust for individual time-invariant covariates, including age, sex, and habitual risk factors.^19,20^ The case day was defined as the date of AMI admission, while the control day was a day when AMI was assumed not to have occurred. Control days were selected from the same days of the week as the case day within the same month and year to account for seasonality, exposure trend bias, and potential confounders. This selection method minimized the risk of overlapping exposures. In this time-stratified case-crossover design, control days were selected from the same day of the week as the case day, within the same month and year but excluding the same week, to account for seasonality and potential confounders, including long-term temporal trends. This approach helps reduce the risk of overlapping exposures and bias.

### Air pollutants and environmental data

Meteorological data, including the ambient temperature and relative humidity, as well as air pollutant data, including nitrogen dioxide (NO_2_), sulphur dioxide (SO_2_), PM_2.5_, and photochemical oxidants (Ox), were sourced from the Kankyotenbodai, published in Japanese by the National Institute for Environmental Studies.^21^ All data were measured on an hourly basis. From these, 24-h mean values were calculated for temperature, humidity, NO_2_, SO_2_, and PM_2.5_, while the 24-h maximum concentration was used for Ox. Individual exposure levels were estimated using data from the monitoring station closest to the admitting hospital. A summary of the number of stations and hospital–station distances (median and interquartile range [IQR]) for each parameter is provided in Supplementary data online, *Table S1*.

### Statistical analysis

Continuous variables were presented as medians with IQRs, while categorical variables as number and percentages. ORs with 95% confidence intervals (CIs) of AMI risk associated with short-term exposure to PM_2.5_ were calculated per 10 μg/m³ increase in PM_2.5_ using a conditional logistic model. To assess the acute effects of short-term PM_2.5_ exposure, we examined single-day lag models ranging from lag 0 (same day exposure) to lag 5 (exposure 5 days prior). The lag-2 day model showed the strongest and statistically significant association with AMI risk, and was therefore selected for the main analysis (see Supplementary data online, *Figure S2*). The lag-2 day effect of PM_2.5_ was statistically significant when combining data across all seasons. This observation was also supported by previous literature reporting that the cardiovascular effects of PM_2.5_ exposure peak approximately 2 days after exposure.^12^ However, in season-stratified analyses, this risk elevation remained significant only in spring and was not statistically significant in the other seasons (see Supplementary data online, *Figures S2* and *S3*). Nevertheless, to provide a more stable and generalizable estimate of the overall public health risk attributable to PM_2.5_ exposure throughout the year, we defined the all-season analysis as our primary focus. As covariates, natural cubic splines of meteorological parameters (ambient temperature and relative humidity) from the index day to 2 days prior (Day 02), with three knots, were adjusted in the multivariable analysis using a single-pollutant model. In addition, to distinguish the discrete effect of the pandemic from gradual, secular changes in risk, the model was simultaneously adjusted for long-term temporal trends. We incorporated region-specific annual average PM_2.5_ concentrations using a natural cubic spline, which allowed for flexible, non-linear adjustment. The final model, therefore, included interaction terms for short-term PM_2.5_ with both a binary pandemic-period indicator and the continuous long-term trend spline. Pandemic-related behavioural changes were further accounted for using daily prefecture-level mobility indicator such as workplace, transit stations, or residential areas from Google COVID-19 Community Mobility Reports (see Supplementary data online, *Figures S4*).^22^ For the two-pollutant models, PM_2.5_ was the main exposure, with additional adjustment for other air pollutants including NO₂, SO₂, and Ox. In the primary analysis, we classified the study period into pre-pandemic and post-pandemic phases based on 7 April 2020, when the state of emergency was declared. To assess the robustness of the findings, we conducted sensitivity analyses including: (i) alternative exposure assignment using the monitoring station nearest to each patient's residential zip code, (ii) exclusion of hospitals located ≥50 km from the nearest monitoring station, and (iii) an alternative classification using 1 March 2020, and 16 April 2020, as the reference date. In addition, we assessed the robustness of our adjustment for behavioural changes by conducting sensitivity analyses with alternative mobility indices, including models that further adjusted for transit stations or residential mobility in addition to workplace mobility.

The absolute risk difference (ARD) in admissions for AMI, MI-CAD, and MINOCA attributable to PM_2.5_ was calculated as previously described.^6^


ARD=incidencerate×(relativerisk–1)/relativerisk

Since the present study did not include the incidence rates of AMI, MI-CAD, or MINOCA, the age-adjusted hospitalization rate for AMI was assumed to be 43.1 per 100 000 person-years, based on previous report.^23^ The ARD was then estimated using the relative risk, which can be approximated by the OR when the incidence rate is very low.

The population attributable fraction (PAF) using ORs was calculated as previously described.^24^ To approximate the PAF, ORs were treated as relative risks, a common approach when the outcome of interest is rare.

The PAF was calculated using the following formula:


PAF=P×(relativerisk-1)/[P×(relativerisk-1)+1]

where *P* denotes the proportion of the population exposed to the risk factor. This approach aligns with established epidemiological methodologies for estimating PAF based on exposure prevalence and risk estimates.^24^

Subgroup analyses were performed to assess whether the association between short-term PM_2.5_ and myocardial infarction subtypes was modified by baseline characteristics, including age (≥75 years), sex, smoking status, diabetes mellitus (DM), and ability to perform activities of daily living (ADL). For each characteristic, we incorporated a three-way interaction term (PM_2.5_ × Period × Subgroup variable) into the main conditional logistic regression model. The statistical significance of these interaction terms was evaluated using Wald tests. A two-sided *P*-value <.05 was considered statistically significant. All statistical analyses were conducted using SAS9.4 (SAS Institute, Cary, NC, USA) and R software version 4.0.5. (R Foundation for Statistical Computing, Vienna, Austria. https://www.R-project.org/).

## Results

### Patient characteristics and environmental data

Among the 480 806 AMI patients, 247 054 (91.5%) were identified as MI-CAD and 23 037 (8.5%) as MINOCA. The clinical characteristics of the patients are summarized in Supplementary data online, *Table S2*. In the MI-CAD group, the median age was 70 years, 75.5% were male, the median body mass index (BMI) was 23.6 kg/m^2^, and 47.9% were smokers. In the MINOCA group, the median age was 71 years, 65.1% were male, the median BMI was 23.0 kg/m^2^, and 40.1% were smokers.

The patient characteristics for the pre-pandemic and post-pandemic groups are summarized in *Table 1*. The proportion of patients with MINOCA remained relatively stable between the two periods, at 8.6% and 8.4%, respectively. While the median age, proportion of male patients, and median BMI showed no significant differences, the prevalence of dyslipidemia, DM, atrial fibrillation, and renal disease exhibited an increasing trend. In contrast, the prevalence of peripheral vascular disease and low ADL status demonstrated a decreasing trend.

**Table 1:** Descriptive clinical characteristics of AMI, MI-CAD, and MINOCA cases before and after the COVID-19 pandemic

	AMI *n* = 270 091		MI-CAD *n* = 247 054		MINOCA *n* = 23 037	
	Pre-pandemic *n* = 207 487 (77%)	Post-pandemic *n* = 62 604 (23%)	Pre-pandemic *n* = 189 687 (77%)	Post-pandemic *n* = 57 367 (23%)	Pre-pandemic *n* = 17 800 (77%)	Post-pandemic *n* = 5237 (23%)
MINOCA, *n* (%)	17 800 (8.6%)	5237 (8.4%)	–	–	–	–
MI-CAD, *n* (%)	189 687 (91.4%)	57 367 (91.6%)	–	–	–	–
Age, median (IQR)	70 (61–79)	72 (61–80)	70 (61–79)	72 (61–80)	71 (60–80)	72 (60–81)
≥20 to <50 years, *n* (%)	18 040 (8.7)	4677 (7.5)	16 241 (8.6)	4148 (7.2)	1799 (10.1)	529 (10.1)
≥50 to <65	51 881 (25.0)	14 690 (23.5)	47 765 (25.2)	13 532 (23.6)	4116 (23.1)	1158 (22.1)
≥65 to <75	60 483 (29.2)	17 596 (28.1)	55 757 (29.4)	16 280 (28.4)	4726 (26.6)	1316 (25.1)
≥75	77 073 (37.2)	25 640 (41.0)	69 917 (36.9)	23 406 (40.8)	7156 (40.2)	2234 (42.7)
Males, *n* (%)	154 944 (74.7)	46 516 (74.3)	143 307 (75.6)	43 167 (75.3)	11 637 (65.4)	3349 (64.0)
Body mass index, kg/m^2^, median (IQR)	23.6 (21.3–26.0)	23.6 (21.4–26.1)	23.6 (21.4–26.0)	23.7 (21.5–26.3)	22.9 (20.6–25.6)	23.1 (20.8–25.6)
Body mass index, not recorded, *n* (%)	20 147 (7.5)	17 966 (7.3)	14 062 (7.4)	3904 (6.8)	1697 (9.5)	484 (9.2)
Smoking, *n* (%)	99 513 (48.0)	28 044 (44.8)	92 219 (48.6)	26 091 (45.5)	7294 (41.0)	1953 (37.3)
Smoking, not recorded, *n* (%)	24 509 (11.8)	8571 (13.7)	22 185 (11.7)	7749 (13.5)	2324 (13.1)	822 (15.7)
Killip classification, *n* (%)						
Killip 1	99 695 (48.0)	30 211 (48.3)	92 495 (49.8)	28 092 (50.1)	7200 (50.9)	2119 (51.8)
Killip 2	50 657 (24.4)	14 483 (23.1)	47 647 (25.7)	13 636 (24.3)	3010 (21.3)	847 (20.7)
Killip 3	16 140 (7.8)	4987 (8.0)	14 925 (8.0)	4644 (8.3)	1215 (8.6)	343 (8.4)
Killip 4	25 039 (12.1)	7399 (11.8)	23 606 (12.7)	7071 (12.6)	1433 (10.1)	328 (8.0)
Unknown	15 956 (7.7)	5524 (8.8)	11 014 (5.8)	3924 (6.8)	4942 (27.8)	1600 (30.6)
Killip ≥3	41 179 (19.8)	12 386 (19.8)	38 531 (21.6)	11 715 (21.9)	2648 (20.6)	671 (18.5)
Cardiac arrest, *n* (%)	7336 (3.5)	2195 (3.5)	6902 (3.6)	2079 (3.6)	434 (2.4)	116 (2.2)
Hypertension, *n* (%)	128 352 (61.9)	38 300 (61.2)	119 828 (63.2)	35 781 (62.4)	8524 (47.9)	2519 (48.1)
Dyslipidaemia, *n* (%)	125 037 (60.3)	39 913 (63.8)	119 216 (62.9)	37 893 (66.1)	5821 (32.7)	2020 (38.6)
Diabetes, *n* (%)	63 769 (30.7)	20 115 (32.1)	59 167 (31.2)	18 772 (32.7)	4602 (25.9)	1343 (25.6)
Atrial fibrillation, *n* (%)	13 040 (6.3)	4826 (7.7)	11 267 (5.9)	4227 (7.4)	1773 (10.0)	599 (11.4)
Chronic pulmonary disease, *n* (%)	5611 (2.7)	1795 (2.9)	4979 (2.6)	1626 (2.8)	632 (3.6)	169 (3.2)
Peripheral vascular disease, *n* (%)	6965 (3.4)	1563 (2.5)	6529 (3.4)	1465 (2.6)	436 (2.5)	98 (1.9)
Cerebrovascular disease, *n* (%)	9675 (4.7)	2751 (4.4)	8602 (4.5)	2449 (4.3)	1073 (6.0)	302 (5.8)
Liver disease, *n* (%)	2602 (1.3)	730 (1.2)	2253 (1.2)	648 (1.1)	349 (2.0)	82 (1.6)
Renal disease, *n* (%)	11 508 (5.6)	4542 (7.3)	10 128 (5.3)	4026 (7.0)	1380 (7.8)	516 (9.9)
Malignancy, *n* (%)	6496 (3.1)	2210 (3.5)	5715 (3.0)	1926 (3.4)	781 (4.4)	284 (5.4)
Low ADL status on admission, *n* (%)	151 327 (72.9)	42 173 (67.4)	139 741 (73.8)	39 065 (68.2)	11 586 (65.3)	3108 (59.5)
ADL status on admission, not recorded, *n* (%)	402 (0.2)	113 (0.2)	349 (0.2)	102 (0.2)	53 (0.3)	11 (0.2)

ADL, activities of daily living; AMI, acute myocardial infarction; IQR, interquartile range; MI-CAD, myocardial infarction with coronary artery disease; MINOCA, myocardial infarction with non-obstructive coronary arteries.

Supplementary data online, *Figure S3* shows a map of Japan, stratified by its 47 prefectures, along with the seasonal variations in mean PM_2.5_ concentrations. PM_2.5_ levels appeared to be higher in spring compared to other seasons, particularly in western Japan (see Supplementary data online, *Figure S5*).

### Main analysis

During the study period, there were 270 091 case days and 916 366 control days in AMI patients, 247 054 and 838 308 in MI-CAD, and 23 037 and 78 058 in MINOCA, respectively (*Table 1*). Each 10-µg/m^3^ increase in PM_2.5_ exposure 2 days prior to admission was associated with hospital admission for AMI (OR 1.105, 95% CI 1.022–1.193, at an annual average PM_2.5_ level of 15 µg/m^3^), MINOCA (OR 1.303, 95% CI 1.005–1.688, at an annual average PM_2.5_ level of 15 µg/m^3^), and MI-CAD (OR 1.088, 95% CI 1.004–1.180, at an annual average PM_2.5_ level of 15 µg/m^3^), after adjusting for meteorological variables and long-term PM_2.5_ trend (*Table 2*). These associations remained statistically significant even after further adjustment for other air pollutants (NO₂, SO₂, or Ox) or mobility index, along with meteorological variables. However, some models did not reach statistical significance (*Table 2*). The ARD of AMI associated with short-term increase of 10-µg/m³ in PM_2.5_ 2 days before admission was 4.1 per 100 000 person-years, while that for MINOCA and MI-CAD was 0.9 and 3.2, respectively (*Table 2*).

**Table 2:** Association between short-term exposure to PM_2.5_ and lag-2 day admission for AMI and subtypes, adjusted for annual PM_2.5_ trend

	AMI		MI-CAD		MINOCA	
	OR (95% CI)	ARD, no. per 100 000 at risk per year (95% CI)	OR (95% CI)	ARD, no. per 100 000 at risk per year (95% CI)	OR (95% CI)	ARD, no. per 100 000 at risk per year (95% CI)
An annual average PM_2.5_ level of 10 μg/m^3^						
Meteorological variables	1.108 (1.023–1.200)	4.2 (1.0–7.2)	1.091 (1.003–1.186)	3.3 (0.1–6.2)	1.314 (1.005–1.719)	0.9 (0.0–1.5)
Meteorological variables + NO_2_ (Day 02)	1.106 (1.019–1.201)	4.1 (0.8–7.2)	1.091 (1.001–1.189)	3.3 (0.0–6.3)	1.288 (0.977–1.698)	0.8 (−0.1–1.5)
Meteorological variables + SO_2_ (Day 02)	1.110 (1.020–1.207)	4.3 (0.9–7.4)	1.091 (0.999–1.191)	3.3 (0.0–6.3)	1.334 (1.007–1.768)	0.9 (0.0–1.6)
Meteorological variables + Ox (Day 02)	1.117 (1.029–1.211)	4.5 (1.2–7.5)	1.098 (1.008–1.196)	3.5 (0.3–6.5)	1.332 (1.013–1.751)	0.9 (0.0–1.6)
Meteorological variables + mobility index	1.105 (1.020–1.196)	4.1 (0.8–7.1)	1.087 (1.000–1.182)	3.2 (0.0–6.1)	1.310 (1.001–1.713)	0.8 (0.0–1.5)
An annual average PM_2.5_ level of 15 μg/m^3^						
Meteorological variables	1.105 (1.022–1.193)	4.1 (0.9–7.0)	1.088 (1.004–1.180)	3.2 (0.1–6.0)	1.303 (1.005–1.688)	0.9 (0.0–1.5)
Meteorological variables + NO_2_ (Day 02)	1.103 (1.018–1.194)	4.0 (0.8–7.0)	1.088 (1.001–1.183)	3.2 (0.0–6.1)	1.277 (0.978–1.668)	0.8 (−0.1–1.5)
Meteorological variables + SO_2_ (Day 02)	1.106 (1.020–1.200)	4.1 (0.8–7.2)	1.088 (0.999–1.185)	3.2 (0.0–6.1)	1.322 (1.007–1.735)	0.9 (0.0–1.6)
Meteorological variables + Ox (Day 02)	1.113 (1.029–1.204)	4.4 (1.2–7.3)	1.095 (1.009–1.190)	3.4 (0.3–6.3)	1.320 (1.013–1.719)	0.9 (0.0–1.5)
Meteorological variables + mobility index	1.101 (1.020–1.189)	4.0 (0.8–6.9)	1.085 (1.000–1.176)	3.1 (0.0–5.9)	1.298 (1.002–1.683)	0.8 (0.0–1.5)
An annual average PM_2.5_ level of 20 μg/m^3^						
Meteorological variables	1.109 (1.023–1.202)	4.2 (1.0–7.2)	1.091 (1.003–1.187)	3.3 (0.1–6.2)	1.316 (1.004–1.725)	0.9 (0.0–1.5)
Meteorological variables + NO_2_ (Day 02)	1.107 (1.019–1.202)	4.2 (0.8–7.3)	1.091 (1.001–1.191)	3.3 (0.0–6.3)	1.290 (0.976–1.704)	0.8 (−0.1–1.5)
Meteorological variables + SO_2_ (Day 02)	1.111 (1.020–1.209)	4.3 (0.9–7.4)	1.091 (0.999–1.193)	3.3 (−0.1–6.4)	1.336 (1.006–1.775)	0.9 (0.0–1.6)
Meteorological variables + Ox (Day 02)	1.117 (1.029–1.213)	4.5 (1.2–7.6)	1.099 (1.008–1.198)	3.5 (0.3–6.5)	1.334 (1.013–1.757)	0.9 (0.0–1.6)
Meteorological variables + mobility index	1.105 (1.020–1.198)	4.1 (0.8–7.1)	1.088 (1.000–1.183)	3.2 (0.0–6.1)	1.311 (1.000–1.719)	0.9 (0.0–1.5)

AMI, acute myocardial infarction; ARD, absolute risk difference; CI, confidence interval; MI-CAD, myocardial infarction with coronary artery disease; MINOCA, myocardial infarction with nonobstructive coronary arteries; NO_2_, nitrogen dioxide; OR, odds ratio; Ox, photochemical oxidant; SO_2_, sulphur dioxide.

ORs and 95% CIs of short-term exposure to PM_2.5_ were adjusted for natural cubic splines of temperature (Day 02), humidity (Day 02), and annual average levels of PM_2.5_ with three knots in the single-pollutant model. In the two-pollutant model, PM_2.5_ was the primary exposure, and additional pollutants (NO_2_, SO_2_, or Ox) were included in the model to assess their confounding effects. An additional multivariable model using a workplace-related mobility index as a covariate was employed to adjust for behavioural changes induced by the COVID-19 pandemic.

The ARD was calculated based on the age-adjusted hospitalization rate for AMI that was 43.1 per 100 000 person-years in 2011 in Kumamoto, Japan.

PAFs of AMI, MINOCA, and MI-CAD associated with a short-term increase in PM_2.5_ 2 days before admission are presented in *Figure 1*. When the exposure prevalence was 1.0, the PAFs were 9.47% for AMI, 8.09% for MI-CAD, and 23.2% for MINOCA.

**Figure 1:**
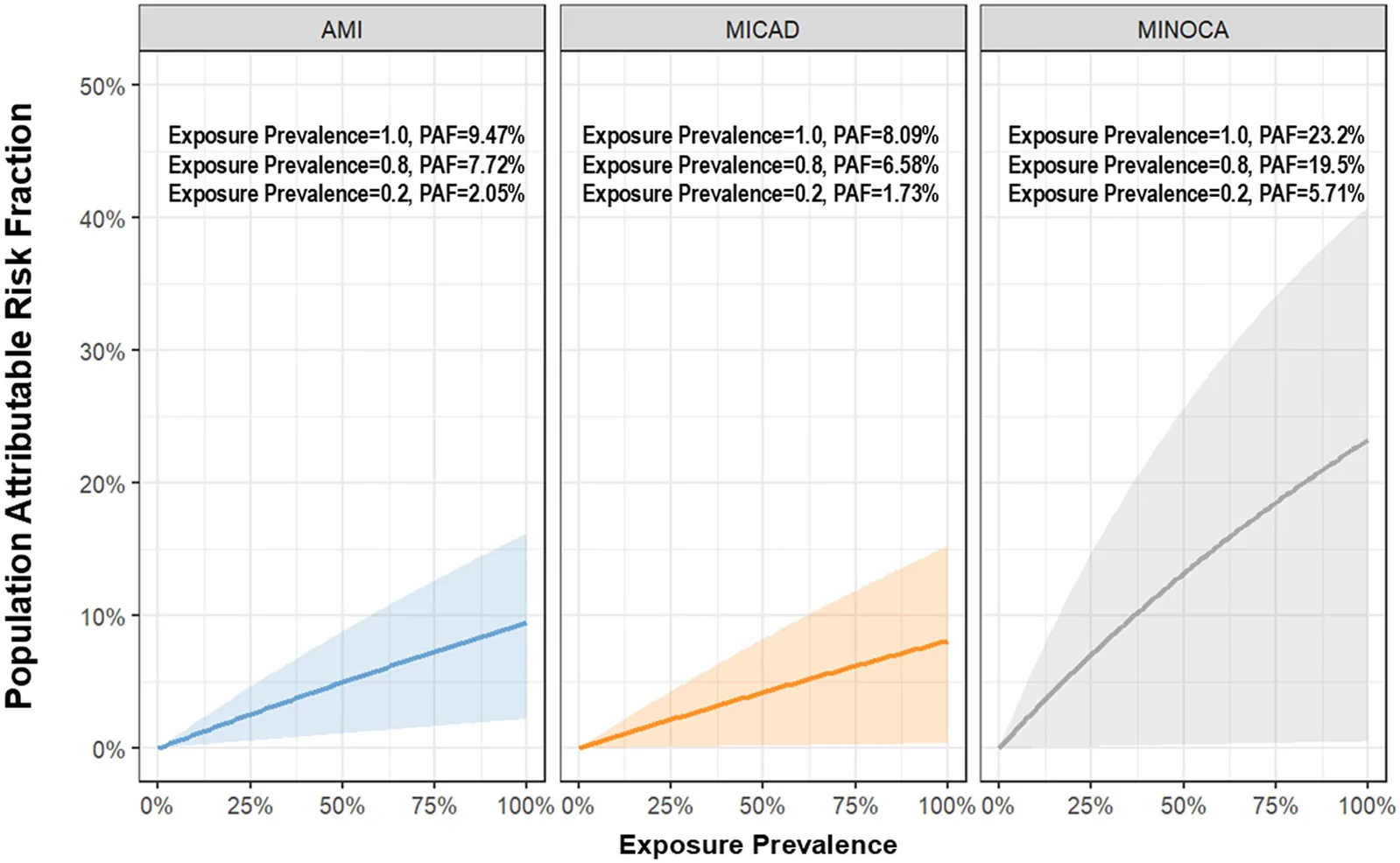
Population attributable risk fraction of AMI, MINOCA, and MI-CAD associated with short-term exposure to PM_2.5_ two days before admission in all seasons. The solid line presents the estimated population attributable risk fraction (PAF) of AMI, MINOCA, and MI-CAD associated with short-term exposure to PM_2.5_, with the shaded area indicating the 95% confidence interval. The PAF represents the estimated proportion of AMI events at the population level that are associated with PM2.5 exposure above the reference level, based on the observed exposure–risk relationship. This measure reflects population-level impact and should not be interpreted as an estimate of individual-level risk. The x-axis represents exposure prevalence, while the y-axis indicates the PAF. AMI, acute myocardial infarction; PM_2.5_, particulate matter with an aerodynamic diameter ≤2.5 µm; MI-CAD, myocardial infarction with coronary artery disease; MINOCA, myocardial infarction with non-obstructive coronary arteries

*Figure 2* shows the association between short-term PM_2.5_ exposure and MI subtypes, stratified by the pre- and post-pandemic periods across a range of long-term annual PM_2.5_ trends. In the primary model adjusted for meteorological variables, the effect of short-term PM_2.5_ exposure was significantly attenuated during the post-pandemic period for MINOCA (*P* for interaction = .017), but not for AMI (*P* for interaction = .070) or MI-CAD (*P* for interaction = .241). These period-specific associations remained largely consistent after further adjustment for co-pollutants or mobility index (*Table 3*). Across all models, the interaction between short-term PM_2.5_ exposure and the long-term PM_2.5_ trend was not statistically significant for any of the MI subtypes.

**Figure 2:**
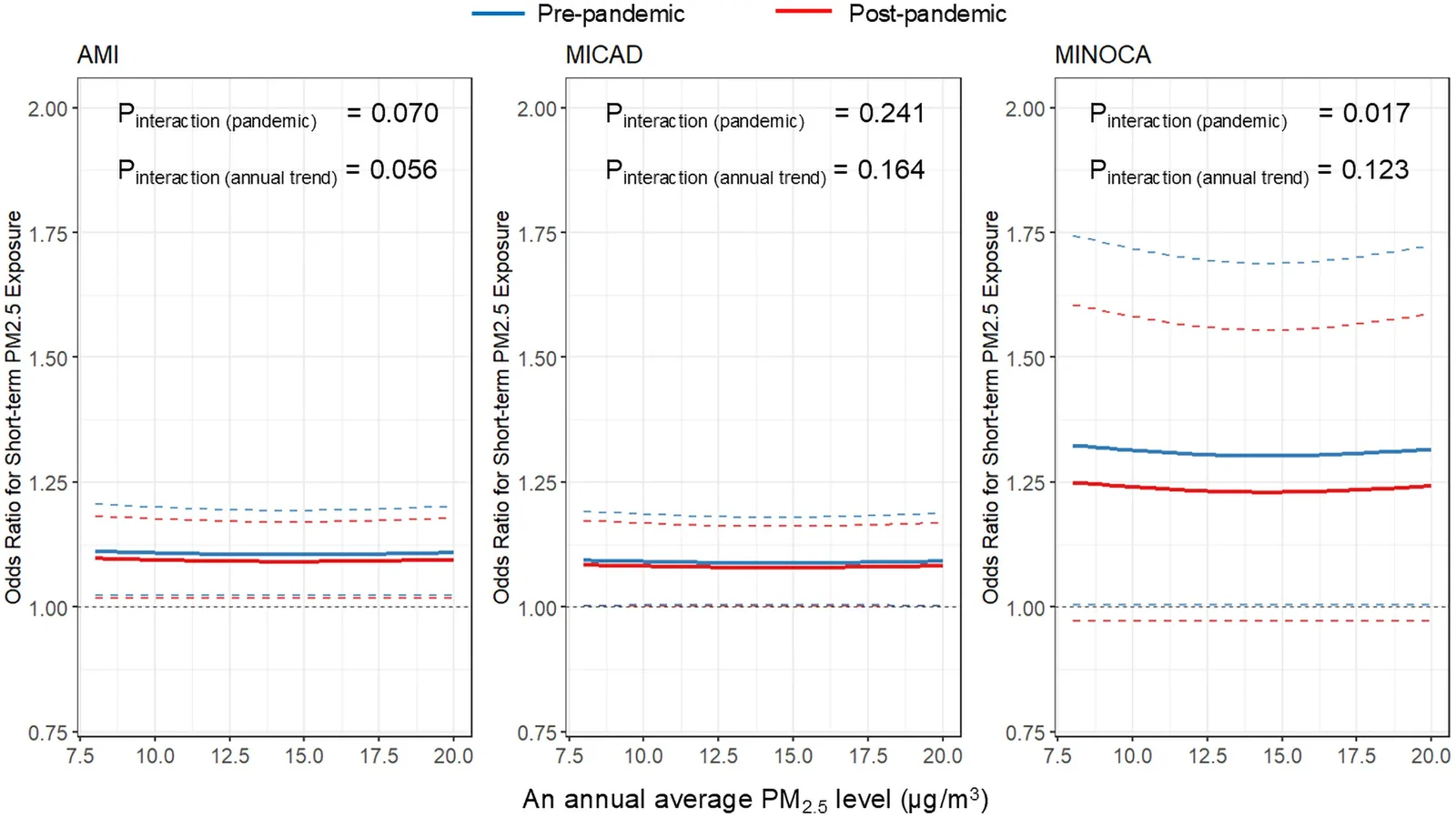
Association between short-term PM_2.5_ exposure and AMI, MINOCA, and MI-CAD, stratified by pandemic period and long-term PM_2.5_ trend. Odds ratios and 95% confidence intervals of short-term exposure to PM_2.5_ were adjusted for natural cubic splines of temperature (Day 2), and humidity (Day 2) with three knots, and annual average levels of PM_2.5_ with three knots, stratified by pre-pandemic and post-pandemic periods. While the association did not significantly vary by the long-term PM₂.₅ trend (*x*-axis) for any subtype, a significant interaction by pandemic period was found for MINOCA (*P* = .017), with a lower OR in the post-pandemic period. No such interaction was observed for AMI (*P* = .070) or MI-CAD (*P* = .241). AMI, acute myocardial infarction; PM_2.5_, particulate matter with an aerodynamic diameter ≤2.5 µm; MI-CAD, myocardial infarction with coronary artery disease; MINOCA, myocardial infarction with non-obstructive coronary arteries

**Table 3:** Interaction analysis of short-term PM_2.5_ exposure on acute myocardial infarction and subtypes before vs. during the COVID-19 pandemic

	AMI		MI-CAD		MINOCA	
	OR (95% CI)	*P* for interaction	OR (95% CI)	*P* for interaction	OR (95% CI)	*P* for interaction
Meteorological variables						
Pre-pandemic	1.105 (1.022–1.193)	.070	1.088 (1.004–1.180)	.241	1.303 (1.005–1.688)	.017
Post-pandemic	1.091 (1.017–1.170)		1.079 (1.002–1.161)		1.230 (0.973–1.555)	
Meteorological variables + NO_2_ (Day 02)						
Pre-pandemic	1.103 (1.018–1.194)	.056	1.088 (1.001–1.183)	.241	1.277 (0.978–1.668)	.006
Post-pandemic	1.088 (1.012–1.169)		1.078 (1.000–1.163)		1.193 (0.937–1.519)	
Meteorological variables + SO_2_ (Day 02)						
Pre-pandemic	1.106 (1.020–1.200)	.101	1.088 (0.999–1.185)	.309	1.322 (1.007–1.735)	.023
Post-pandemic	1.093 (1.015–1.177)		1.080 (0.999–1.166)		1.247 (0.975–1.595)	
Meteorological variables + Ox (Day 02)						
Pre-pandemic	1.113 (1.029–1.204)	.082	1.095 (1.009–1.190)	.254	1.320 (1.013–1.719)	.026
Post-pandemic	1.099 (1.023–1.181)		1.086 (1.007–1.171)		1.250 (0.984–1.588)	
Meteorological variables + mobility index						
Pre-pandemic	1.101 (1.020–1.189)	.073	1.085 (1.000–1.176)	.248	1.298 (1.002–1.683)	.018
Post-pandemic	1.088 (1.014–1.166)		1.076 (0.999–1.158)		1.226 (0.970–1.551)	

AMI, acute myocardial infarction; CI, confidence interval; MI-CAD, myocardial infarction with coronary artery disease; MINOCA, myocardial infarction with nonobstructive coronary arteries; NO_2_, nitrogen dioxide; OR, odds ratio; Ox, photochemical oxidant; SO_2_, sulphur dioxide.

ORs and 95% CIs of short-term exposure to PM_2.5_ were adjusted for natural cubic splines of temperature (Day 02), humidity (Day 02), and annual average levels of PM_2.5_ with three knots in the single-pollutant model. In the two-pollutant model, PM_2.5_ was the primary exposure, and additional pollutants (NO_2_, SO_2_, or Ox) were included in the model to assess their confounding effects. An additional multivariable model using a workplace-related mobility index as a covariate was employed to adjust for behavioural changes induced by the COVID-19 pandemic.

### Sensitivity analysis

Sensitivity analyses including residential municipal code analysis, distance ≤50 km between the hospital and monitoring site analysis, and an alternative reference date analysis using 1 March 2020 and 16 April 2020 as the reference dates showed a significant decrease in ORs for MINOCA after COVID-19 pandemic (all *P* for interaction <.05), consistent with the main results (*Table 4*). The results of sensitivity analyses using alternative mobility indices were consistent with our primary findings. The statistically significant interaction between PM_2.5_ exposure and the pandemic period for MINOCA remained robust across all tested mobility-adjusted models (see Supplementary data online, *Table S3*).

**Table 4:** Sensitivity analysis of association between short-term exposure to PM2.5 two days before admission and admission for MINOCA before and after COVID-19 pandemic

	Residential municipal code analysis		Distance ≤ 50 km between the hospital and monitoring site		Alternative Reference Date (1 March 2020)		Alternative Reference Date (16 April 2020)	
	OR (95% CI)	*P* for interaction	OR (95% CI)	*P* for interaction	OR (95% CI)	*P* for interaction	OR (95% CI)	*P* for interaction
Meteorological variables		<.001		.016		.018		.018
Pre-pandemic	1.254 (0.921–1.709)		1.305 (1.007–1.692)		1.304 (1.005–1.691)		1.302 (1.005–1.687)	
Post-pandemic	1.128 (0.854–1.490)		1.232 (0.974–1.557)		1.232 (0.974–1.559)		1.230 (0.973–1.554)	
Meteorological variables + NO_2_ (Day 02)		.001		.006		.006		.006
Pre-pandemic	1.202 (0.876–1.648)		1.279 (0.979–1.670)		1.283 (0.982–1.677)		1.279 (0.980–1.669)	
Post-pandemic	1.085 (0.816–1.442)		1.195 (0.938–1.522)		1.198 (0.940–1.527)		1.194 (0.938–1.521)	
Meteorological variables + SO_2_ (Day 02)		.004		.022		.024		.020
Pre-pandemic	1.228 (0.889–1.694)		1.326 (1.010–1.740)		1.324 (1.008–1.740)		1.326 (1.011–1.740)	
Post-pandemic	1.119 (0.837–1.496)		1.250 (0.977–1.599)		1.250 (0.977–1.601)		1.250 (0.978–1.597)	
Meteorological variables + Ox (Day 02)		<.001		.025		.028		.025
Pre-pandemic	1.278 (0.933–1.749)		1.323 (1.016–1.724)		1.322 (1.014–1.723)		1.323 (1.016–1.722)	
Post-pandemic	1.139 (0.859–1.512)		1.253 (0.986–1.591)		1.253 (0.985–1.592)		1.253 (0.986–1.591)	
Meteorological variables + mobility index		<.001		.018		.020		.019
Pre-pandemic	1.237 (0.908–1.686)		1.301 (1.004–1.687)		1.297 (1.000–1.683)		1.298 (1.002–1.682)	
Post-pandemic	1.115 (0.844–1.473)		1.228 (0.971–1.554)		1.227 (0.969–1.552)		1.227 (0.971–1.551)	

AMI, acute myocardial infarction; CI, confidence interval; MI-CAD, myocardial infarction with coronary artery disease; MINOCA, myocardial infarction with nonobstructive coronary arteries; NO_2_, nitrogen dioxide; OR, odds ratio; Ox, photochemical oxidant; SO_2_, sulfur dioxide.

ORs and 95% CIs of short-term exposure to PM_2.5_ were adjusted for natural cubic splines of temperature (Day 02), humidity (Day 02), and annual average levels of PM_2.5_ with three knots in the single-pollutant model. In the two-pollutant model, PM_2.5_ was the primary exposure, and additional pollutants (NO_2_, SO_2_, or Ox) were included in the model to assess their confounding effects. An additional multivariable model using a workplace-related mobility index as a covariate was employed to adjust for behavioural changes induced by the COVID-19 pandemic.

### Subgroup analysis

*Figure 3* shows the results of the subgroup analyses for the association between short-term PM_2.5_ exposure and MI subtypes, evaluated at an annual average PM_2.5_ level of 15 µg/m^3^. We found no evidence of statistically significant effect modification by any of the patient characteristics, including age, sex, smoking habit, diabetes mellitus, and ADL status, for any of the MI subtypes. This lack of a significant interaction was observed consistently in both the pre-pandemic and post-pandemic periods (*Figure 3*).

**Figure 3:**
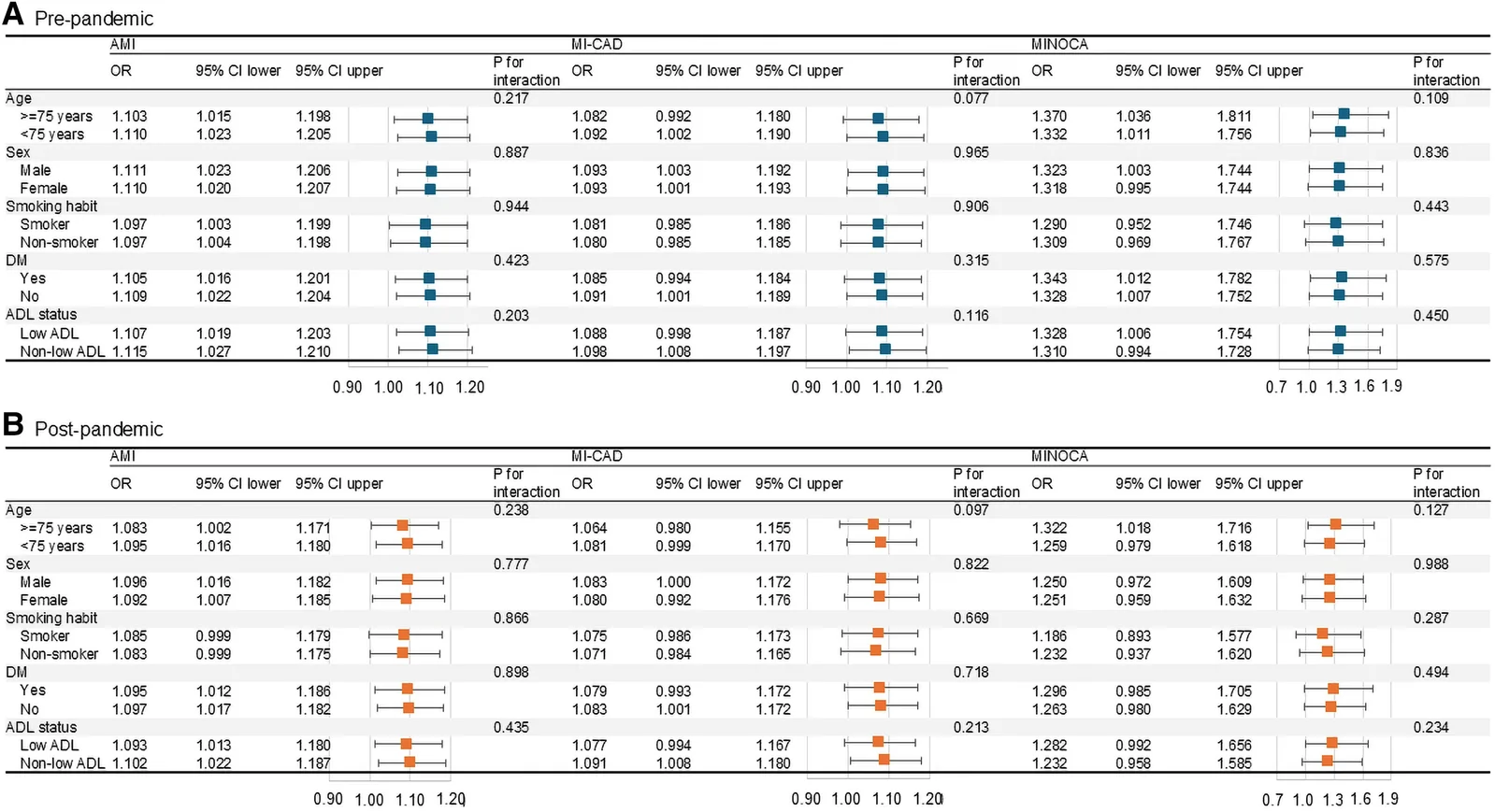
Subgroup analysis of the association between short-term exposure to PM_2.5_ two days before admission and admission for AMI, MINOCA, and MI-CAD before and after COVID-19 pandemic. Odds ratios and 95% confidence intervals of short-term exposure to PM_2.5_ were adjusted for natural cubic splines of temperature (Day 2), and humidity (Day 2) with three knots, and annual average levels of PM_2.5_ with three knots in the single-pollutant model. ADL, activities of daily living; AMI, acute myocardial infarction; DM, diabetes mellitus; PM_2.5_, particulate matter with an aerodynamic diameter ≤2.5 µm; MI-CAD, myocardial infarction with coronary artery disease; MINOCA, myocardial infarction with non-obstructive coronary arteries

## Discussion

In this nationwide study, we investigated the association between short-term exposure to PM_2.5_ and the risk of AMI and its subtypes, MI-CAD and MINOCA, using a natural experimental design that employed the COVID-19 pandemic as an exogenous intervention. Our findings indicated that each 10-µg/m³ increase in PM_2.5_ exposure 2 days prior to admission was associated with a high risk of hospital admission for AMI and the subtypes. Importantly, although the risk estimates for AMI overall and MI-CAD remained relatively stable before and after the pandemic, a significant attenuation in the OR for MINOCA was observed in the post-pandemic period (pre-pandemic OR 1.303, 95% CI 1.005–1.688; post-pandemic OR 1.230, 95% CI 0.973–1.555; *P* for interaction = .017), with an observation that was further supported by sensitivity analyses (*Structured Graphical Abstract*).

The differential change in ORs for MINOCA observed before and after the pandemic suggested that public health measures, such as mask-wearing, mobility restrictions, and other behavioural modifications, may have modified individual exposures to PM_2.5_ or altered the underlying pathophysiological pathways leading to MINOCA. In particular, coronary spasm and microvascular dysfunction are mediated by oxidative stress-induced endothelial dysfunction and chronic low-grade inflammation of vascular smooth muscle.^25–27^ Bevan *et al*. further demonstrated that PM_2.5_ exposure accelerates atherosclerosis and endothelial dysfunction through oxidative and inflammatory pathways, providing mechanistic insights into pollution-driven cardiovascular risk.^28^ Basic studies have demonstrated that air pollutants such as PM_2.5_, SO₂, and NO₂ induce the production of reactive oxygen species (ROS), which in turn contribute to vascular injury,^29–32^ and human studies have confirmed that such exposures impair endothelial function.^33^ Given that MINOCA is characterized by heterogeneous mechanisms including microvascular dysfunction and coronary spasm,^2,4,34,35^ it is plausible that reductions in ambient PM_2.5_ exposure or improved protective behaviours during the pandemic could have preferentially attenuated these specific mechanisms. This idea is supported by prior studies that have characterized the multifactorial pathogenesis of MINOCA^36,37^ and by recent analyses reporting significant reductions in air pollutant levels during pandemic-related lockdowns.^38,39^ However, Japan's baseline PM_2.5_ levels were not inherently high, and thus the reductions in ambient PM_2.5_ concentrations observed during the COVID-19 pandemic were modest, unlike those seen in countries with significantly higher pollution levels. The magnitude and persistence of these changes likely would varied by region and calendar period and would be strongly influenced by seasonality and long-term temporal trends. Therefore, our findings should not be interpreted as evidence that large, uniform reductions in ambient PM_2.5_ concentrations occurred during the pandemic. Moreover, the relatively modest behavioural restrictions in Japan compared to countries with mandatory lockdowns^40^ suggest that the pandemic-related attenuation in PM_2.5_-associated MINOCA risk may not have been primarily driven by reductions in ambient exposure due to limited mobility or activity. While changes in population movement were accounted for using Google COVID-19 Community Mobility Reports,^22^ the observed reduction in risk persisted after this adjustment. This finding implies that factors beyond mobility may have contributed to attenuating PM_2.5_ inhalation and its downstream cardiovascular effects. Among these factors, widespread mask wearing, which was rapidly adopted and consistently practiced in Japan even without legal mandates, likely played a particularly important role. Thus, our results may indirectly highlight the protective role of non-pharmaceutical interventions, particularly face masks, in reducing acute cardiovascular risk associated with air pollution exposure.

Although the absolute differences in risk estimates may appear modest, the clinical and public health implications remain meaningful. Even small relative risks, when applied to common outcomes such as AMI across large populations, may translate into a substantial disease burden. Furthermore, the specific attenuation observed in MINOCA, a subtype associated with mechanisms such as coronary spasm and microvascular dysfunction, suggests a biologically plausible and selectively modifiable risk pathway. Therefore, our findings not only demonstrate epidemiological associations but also contribute to hypothesis generation regarding vulnerable cardiovascular phenotypes that may respond differently to environmental interventions.

Our findings align with previous epidemiological studies demonstrating that short-term exposure to PM_2.5_ is associated with a high risk of cardiovascular events.^12,13,41–44^ Earlier investigations have highlighted seasonal variations in PM_2.5_ concentrations and their corresponding cardiovascular risks, with spring exposures often being related to higher risk possibly due to transboundary pollution and specific seasonal sources.^12,13,45,46^ In addition to seasonal variations in PM_2.5_ concentrations, temperature could also play a crucial role in influencing its effects. Recent study reported that MINOCA is more susceptible to low ambient temperature compared to MI-CAD,^47^ thereby suggesting that environmental triggers may differ between AMI subtypes. In the present study, we observed a significant reduction in the OR for PM_2.5_-related risk of MINOCA after the pandemic, whereas no such reduction was evident for those of MI-CAD. As shown in Supplementary data online, *Figure S5*, PM_2.5_ concentrations have gradually declined over time, and at these lower levels, the association between short-term exposure to PM_2.5_ and MI-CAD was not statistically significant.^12^ Consequently, the minimal difference in ORs for MI-CAD between the pandemic periods may be attributed to the lower PM_2.5_ concentrations in our study region. Had the study been conducted in an area with higher PM_2.5_ levels, the difference in ORs before and after the pandemic might have been more evident. Nevertheless, because the progression of atherosclerosis in MI-CAD is also mediated by oxidative stress and endothelial dysfunction,^48^ substantial benefits from reducing PM_2.5_ exposure remain plausible. Future research in regions with higher PM_2.5_ concentrations is warranted to further validate these findings.

By adopting a natural experimental framework with the COVID-19 pandemic as an exogenous intervention, we were able to assess potential modifications in the exposure–outcome relationship under real-world conditions, thereby strengthening causal inference.^17,18^ Despite this strength, several limitations warrant consideration. First, the observational nature of our study inherently limits causal interpretation, and residual confounding from unmeasured factors cannot be entirely ruled out. Second, exposure assessment was based on ambient pollutant concentrations from monitoring stations nearest to the admitting hospitals, which may not accurately capture individual-level exposures, especially in the context of indoor air quality and variable personal behaviours during the pandemic. However, to address this potential limitation, we conducted sensitivity analyses, including an alternative exposure assignment using the station nearest to each patient's residential zip code and an analysis excluding hospitals located ≥50 km from the nearest monitor. The results of these analyses were consistent with our primary findings, supporting the robustness of our conclusions. Third, although our algorithm for identifying MINOCA indicates high specificity,^12^ its low sensitivity may have led to an underestimation of the true association between PM_2.5_ and MINOCA. Fourth, while we adjusted for large-scale behavioural shifts using mobility data, we could not account for other unmeasured pandemic-related factors, such as heightened psychological stress or changes in dietary habits, which could act as residual confounders. A recent study has reported an increase in the incidence of stress cardiomyopathy,^49^ and these factors may therefore differentially influence AMI subtypes, potentially acting as unmeasured effect modifiers. Fifth, a further methodological consideration is the statistical instability introduced by simultaneously adjusting for short-term PM_2.5_ exposure and its interaction with the long-term PM_2.5_ trend. While adjusting for this long-term trend was crucial to isolate the abrupt effect of the pandemic, the inherent correlation between daily and annual PM_2.5_ concentrations resulted in significant multicollinearity. This appropriately inflated the standard errors of the effect estimates, leading to wider CIs and reduced statistical precision. We ultimately retained this comprehensive model because we believe that rigorously controlling for the confounding effect of the long-term trend provides a more conservative and statistically valid estimate, despite the trade-off in precision. Finally, although the state of emergency provided a pragmatic threshold to delineate pre- and post-pandemic periods, this binary classification might not fully capture the gradual and regionally heterogeneous changes in air quality and public behaviour during the pandemic.

## Conclusion

Our study provided robust evidence that short-term exposure to PM_2.5_ is associated with an increased risk of AMI and its subtypes. Importantly, the observed attenuation of the risk for MINOCA in the post-pandemic period suggested the potential impact of public health interventions on air pollution-related cardiovascular outcomes. Future research should focus on elucidating the underlying biological mechanisms, refining individual-level exposure assessments, and exploring the long-term cardiovascular benefits of sustained improvements in air quality.

## Supplementary Material

ehag102_Supplementary_Data

## Declarations

### Data Availability

The data underlying this article will be shared on reasonable request to the corresponding author.

## References

[1] Roth GA, Johnson C, Abajobir A, Abd-Allah F, Abera SF, Abyu G, et al Global, regional, and national burden of cardiovascular diseases for 10 causes, 1990 to 2015. J Am Coll Cardiol 2017;70:1–25. 10.1016/j.jacc.2017.04.052 PMC5491406 28527533

[2] Ishii M, Kaikita K, Sakamoto K, Seki T, Kawakami K, Nakai M, et al Characteristics and in-hospital mortality of patients with myocardial infarction in the absence of obstructive coronary artery disease in super-aging society. Int J Cardiol 2020;301:108–13. 10.1016/j.ijcard.2019.09.037 31740139

[3] Otsuka Y, Ishii M, Ikebe S, Miyazaki S, Fujisue K, Tabata N, et al Current overview of myocardial infarction with non-obstructive coronary arteries. J Coron Artery Dis 2024;30:13–20. 10.7793/jcad.30.002

[4] Beltrame JF . Assessing patients with myocardial infarction and nonobstructed coronary arteries (MINOCA). J Intern Med 2013;273:182–5. 10.1111/j.1365-2796.2012.02591.x 22998397

[5] Onuma S, Takahashi J, Shiroto T, Godo S, Hao K, Honda S, et al Characteristics and in-hospital outcomes of patients with myocardial infarction with non-obstructive coronary arteries― insights from the real-world JAMIR database ―. Circ J 2025;89:382–90. 10.1253/circj.CJ-24-0422 39384369

[6] Di Q, Dai L, Wang Y, Zanobetti A, Choirat C, Schwartz JD, et al Association of short-term exposure to air pollution with mortality in older adults. JAMA 2017;318:2446–56. 10.1001/jama.2017.17923 PMC5783186 29279932

[7] Cohen AJ, Brauer M, Burnett R, Anderson HR, Frostad J, Estep K, et al Estimates and 25-year trends of the global burden of disease attributable to ambient air pollution: an analysis of data from the Global Burden of Diseases Study 2015. Lancet 2017;389:1907–18. 10.1016/S0140-6736(17)30505-6 PMC5439030 28408086

[8] Rajagopalan S, Al-Kindi SG, Brook RD. Air pollution and cardiovascular disease: JACC state-of-the-art review. J Am Coll Cardiol 2018;72:2054–70. 10.1016/j.jacc.2018.07.099 30336830

[9] Feng S, Gao D, Liao F, Zhou F, Wang X. The health effects of ambient PM2.5 and potential mechanisms. Ecotoxicol Environ Saf 2016;128:67–74. 10.1016/j.ecoenv.2016.01.030 26896893

[10] Lederer AM, Fredriksen PM, Nkeh-Chungag BN, Everson F, Strijdom H, De Boever P, et al Cardiovascular effects of air pollution: current evidence from animal and human studies. Am J Physiol Heart Circ Physiol 2021;320:H1417–h39. 10.1152/ajpheart.00706.2020 33513082

[11] Xu H, Wen Q, Xu X, Yu D, Liu Z, Zhang C, et al Heme oxygenase-1 protects against PM2.5 induced endothelial dysfunction through inhibition of HIF1α. Environ Toxicol Pharmacol 2023;97:104024. 10.1016/j.etap.2022.104024 36427673

[12] Ishii M, Seki T, Kaikita K, Sakamoto K, Nakai M, Sumita Y, et al Association of short-term exposure to air pollution with myocardial infarction with and without obstructive coronary artery disease. Eur J Prev Cardiol 2021;28:1435–44. 10.1177/2047487320904641 34695220

[13] Ishii M, Seki T, Kaikita K, Sakamoto K, Nakai M, Sumita Y, et al Short-term exposure to desert dust and the risk of acute myocardial infarction in Japan: a time-stratified case-crossover study. Eur J Epidemiol 2020;35:455–64. 10.1007/s10654-020-00601-y 31950372

[14] Yasuda S, Miyamoto Y, Ogawa H. Current status of cardiovascular medicine in the aging society of Japan. Circulation 2018;138:965–7. 10.1161/CIRCULATIONAHA.118.035858 30354536

[15] Nakai M, Iwanaga Y, Sumita Y, Kanaoka K, Kawakami R, Ishii M, et al Validation of acute myocardial infarction and heart failure diagnoses in hospitalized patients with the nationwide claim-based JROAD-DPC database. Circ Rep 2021;3:131–6. 10.1253/circrep.CR-21-0004 PMC7956876 33738345

[16] Ishii M, Tsujita K, Okamoto H, Koto S, Nishi T, Nakai M, et al Resources for cardiovascular healthcare associated with 30-day mortality in acute myocardial infarction with cardiogenic shock. Eur Heart J Open 2022;2:oeab047. 10.1093/ehjopen/oeab047 PMC9242083 35919660

[17] Sekikawa A, Horiuchi BY, Edmundowicz D, Ueshima H, Curb JD, Sutton-Tyrrell K, et al A “natural experiment” in cardiovascular epidemiology in the early 21st century. Heart 2003;89:255–7. 10.1136/heart.89.3.255 PMC1767570 12591821

[18] Craig P, Cooper C, Gunnell D, Haw S, Lawson K, Macintyre S, et al Using natural experiments to evaluate population health interventions: new Medical Research Council guidance. J Epidemiol Community Health 2012;66:1182–6. 10.1136/jech-2011-200375 PMC3796763 22577181

[19] Levy D, Lumley T, Sheppard L, Kaufman J, Checkoway H. Referent selection in case-crossover analyses of acute health effects of air pollution. Epidemiology 2001;12:186–92. 10.1097/00001648-200103000-00010 11246579

[20] Janes H, Sheppard L, Lumley T. Case-crossover analyses of air pollution exposure data: referent selection strategies and their implications for bias. Epidemiology 2005;16:717–26. 10.1097/01.ede.0000181315.18836.9d 16222160

[21] National Institute for Environmental Studies . Kankyotenbodai. https://tenbou.nies.go.jp/download/. Date accessed 11 April 2024.

[22] Google LLC “COVID-19 Community Mobility Reports”. https://www.google.com/covid19/mobility/. Date accessed 22 June 2025.

[23] Kojima S, Matsui K, Ogawa H. Temporal trends in hospitalization for acute myocardial infarction between 2004 and 2011 in Kumamoto, Japan. Circ J 2013;77:2841–3. 10.1253/circj.CJ-13-1011 24067325

[24] Mustafic H, Jabre P, Caussin C, Murad MH, Escolano S, Tafflet M, et al Main air pollutants and myocardial infarction: a systematic review and meta-analysis. JAMA 2012;307:713–21. 10.1001/jama.2012.126 22337682

[25] Crea F, Montone RA, Rinaldi R. Pathophysiology of coronary microvascular dysfunction. Circ J 2022;86:1319–28. 10.1253/circj.CJ-21-0848 34759123

[26] Hubert A, Seitz A, Pereyra VM, Bekeredjian R, Sechtem U, Ong P. Coronary artery spasm: the interplay between endothelial dysfunction and vascular smooth muscle cell hyperreactivity. Eur Cardiol 2020;15:e12. 10.15420/ecr.2019.20 PMC7199189 32373185

[27] Rehan R, Yong A, Ng M, Weaver J, Puranik R. Coronary microvascular dysfunction: a review of recent progress and clinical implications. Front Cardiovasc Med 2023;10:1111721. 10.3389/fcvm.2023.1111721 PMC9908997 36776251

[28] Bevan GH, Al-Kindi SG, Brook RD, Münzel T, Rajagopalan S. Ambient air pollution and atherosclerosis: insights into dose, time, and mechanisms. Arterioscler Thromb Vasc Biol 2021;41:628–37. 10.1161/ATVBAHA.120.315219 33327745

[29] Gutor SS, Salinas RI, Nichols DS, Bazzano JMR, Han W, Gokey JJ, et al Repetitive sulfur dioxide exposure in mice models post-deployment respiratory syndrome. Am J Physiol Lung Cell Mol Physiol 2024;326:L539–l50. 10.1152/ajplung.00239.2023 PMC11380962 38410870

[30] Meng Z, Qin G, Zhang B, Geng H, Bai Q, Bai W, et al Oxidative damage of sulfur dioxide inhalation on lungs and hearts of mice. Environ Res 2003;93:285–92. 10.1016/S0013-9351(03)00045-8 14615239

[31] Jiang J, Liang S, Zhang J, Du Z, Xu Q, Duan J, et al Melatonin ameliorates PM(2.5) -induced cardiac perivascular fibrosis through regulating mitochondrial redox homeostasis. J Pineal Res 2021;70:e12686. 10.1111/jpi.12686 PMC7757260 32730639

[32] Li H, Han M, Guo L, Li G, Sang N. Oxidative stress, endothelial dysfunction and inflammatory response in rat heart to NO₂ inhalation exposure. Chemosphere 2011;82:1589–96. 10.1016/j.chemosphere.2010.11.055 21168897

[33] Zhang H, Yang J, Zhang Y, Xiao K, Wang Y, Si J, et al Age and sex differences in the effects of short- and long-term exposure to air pollution on endothelial dysfunction. Environ Health 2024;23:63. 10.1186/s12940-024-01100-3 PMC11229304 38978038

[34] Severino P, D'Amato A, Prosperi S, Myftari V, Colombo L, Tomarelli E, et al Myocardial infarction with non-obstructive coronary arteries (MINOCA): focus on coronary microvascular dysfunction and genetic susceptibility. J Clin Med 2023;12:3586. 10.3390/jcm12103586 PMC10218901 37240691

[35] Hokimoto S, Kaikita K, Yasuda S, Tsujita K, Ishihara M, Matoba T, et al JCS/CVIT/JCC 2023 guideline focused update on diagnosis and treatment of vasospastic angina (coronary spastic angina) and coronary microvascular dysfunction. Circ J 2023;87:879–936. 10.1253/circj.CJ-22-0779 36908169

[36] Agewall S, Beltrame JF, Reynolds HR, Niessner A, Rosano G, Caforio AL, et al ESC working group position paper on myocardial infarction with non-obstructive coronary arteries. Eur Heart J 2017;38:143–53. 10.1093/eurheartj/ehw149 28158518

[37] Tamis-Holland JE, Jneid H, Reynolds HR, Agewall S, Brilakis ES, Brown TM, et al Contemporary diagnosis and management of patients with myocardial infarction in the absence of obstructive coronary artery disease: a scientific statement from the American Heart Association. Circulation 2019;139:e891–908. 10.1161/CIR.0000000000000670 30913893

[38] Chen K, Wang M, Huang C, Kinney PL, Anastas PT. Air pollution reduction and mortality benefit during the COVID-19 outbreak in China. Lancet Planet Health 2020;4:e210–2. 10.1016/S2542-5196(20)30107-8 PMC7220178 32411944

[39] Muhammad S, Long X, Salman M. COVID-19 pandemic and environmental pollution: a blessing in disguise? Sci Total Environ 2020;728:138820. 10.1016/j.scitotenv.2020.138820 PMC7169881 32334164

[40] Yabe T, Tsubouchi K, Fujiwara N, Wada T, Sekimoto Y, Ukkusuri SV. Non-compulsory measures sufficiently reduced human mobility in Tokyo during the COVID-19 epidemic. Sci Rep 2020;10:18053. 10.1038/s41598-020-75033-5 PMC7581808 33093497

[41] Pope CA, Muhlestein JB, Anderson JL, Cannon JB, Hales NM, Meredith KG, et al Short-term exposure to fine particulate matter air pollution is preferentially associated with the risk of ST-segment elevation acute coronary events. J Am Heart Assoc 2015;4:e002506. 10.1161/JAHA.115.002506 PMC4845284 26645834

[42] Pope CA 3rd, Muhlestein JB, May HT, Renlund DG, Anderson JL, Horne BD. Ischemic heart disease events triggered by short-term exposure to fine particulate air pollution. Circulation 2006;114:2443–8. 10.1161/CIRCULATIONAHA.106.636977 17101851

[43] Kojima S, Michikawa T, Ueda K, Sakamoto T, Matsui K, Kojima T, et al Asian dust exposure triggers acute myocardial infarction. Eur Heart J 2017;38:3202–8. 10.1093/eurheartj/ehx509 29020374

[44] Matsukawa R, Michikawa T, Ueda K, Nitta H, Kawasaki T, Tashiro H, et al Desert dust is a risk factor for the incidence of acute myocardial infarction in western Japan. Circ Cardiovasc Qual Outcomes 2014;7:743–8. 10.1161/CIRCOUTCOMES.114.000921 25074374

[45] Ueda K, Yamagami M, Ikemori F, Hisatsune K, Nitta H. Associations between fine particulate matter components and daily mortality in Nagoya, Japan. J Epidemiol 2016;26:249–57. 10.2188/jea.JE20150039 PMC4848323 26686882

[46] Bell ML, Ebisu K, Peng RD, Walker J, Samet JM, Zeger SL, et al Seasonal and regional short-term effects of fine particles on hospital admissions in 202 US counties, 1999–2005. Am J Epidemiol 2008;168:1301–10. 10.1093/aje/kwn252 PMC2732959 18854492

[47] Huang J, He Q, Jiang Y, Wong JMJ, Li J, Liu J, et al Low ambient temperature and incident myocardial infarction with or without obstructive coronary arteries: a Chinese nationwide study. Eur Heart J 2025;46:439–50. 10.1093/eurheartj/ehae711 39468415

[48] Heitzer T, Schlinzig T, Krohn K, Meinertz T, Münzel T. Endothelial dysfunction, oxidative stress, and risk of cardiovascular events in patients with coronary artery disease. Circulation 2001;104:2673–8. 10.1161/hc4601.099485 11723017

[49] Jabri A, Kalra A, Kumar A, Alameh A, Adroja S, Bashir H, et al Incidence of stress cardiomyopathy during the coronavirus disease 2019 pandemic. JAMA Netw Open 2020;3:e2014780. 10.1001/jamanetworkopen.2020.14780 PMC7348683 32644140

