# Supplementary material for: Air pollution before and during the COVID-19 pandemic: changes in risk of acute myocardial infarction
Source: Eur Heart J. 2026 Feb 13;47(39):5751–64. doi: 10.1093/eurheartj/ehag102 (PMC13690819; doi:10.1093/eurheartj/ehag102)
Supplement: Supplementary file 1 [file ehag102_supplementary_data.docx]

**Supplementary Data Online** for **Air pollution before and during the COVID-19 pandemic: changes in risk of acute myocardial infarction**

Authors: Ishii et al.

**TABLE OF CONTENTS**

**Figure S1**: Study flowchart

**Figure S2**: Association between short-term exposure to air pollutants and admission for AMI, MINOCA, MI-CAD in all seasons

**Figure S3**: PM_2.5_ variation by season in Japan and the association between short-term exposure to PM_2.5_ and admission for AMI, MINOCA, and MI-CAD stratified by season

**Figure S4**: National-average daily mobility patterns in Japan during the COVID-19 pandemic

**Figure S5**: Seasonal variation and monthly trend for PM_2.5_ concentration in Japan

**Table S1**: A Summary of the number of monitoring stations, distance between admitted hospital and the nearest station, and value of data.

**Table S2**: Baseline characteristics of the study population

**Table S3**: Sensitivity analysis using alternative mobility index

**Figure S1**: **Study flowchart**

AMI, acute myocardial infarction; CAG, coronary angiography; JROAD-DPC, Japanese Of All cardiac and vascular Disease-Diagnostic Procedure Combination; MI-CAD, myocardial infarction with coronary artery disease; MINOCA, myocardial infarction with non-obstructive coronary arteries; PM2.5, particulate matter with an aerodynamic diameter ≤2.5 µm; TPNOCA, troponin-positive non-obstructive coronary arteries.

**Figure S2**: **Association between short-term exposure to air pollutants and admission for AMI, MINOCA, MI-CAD in all seasons**

This figure shows the odds ratio and 95% confidence interval of the lag effect of the short-term increase of 10 µg/m^3^ in PM_2.5_ and those of 10-ppb in Ox in all seasons. Odds ratios and 95% confidence intervals were adjusted for natural cubic splines of temperature (Day 02), and humidity (Day 02) with three knots.

AMI, acute myocardial infarction; PM_2.5_, particulate matter with an aerodynamic diameter ≤2.5 µm; MI-CAD, myocardial infarction with coronary artery disease; MINOCA, myocardial infarction with non-obstructive coronary arteries.

**Figure S3: PM_2.5_ variation by season in Japan and the association between short-term exposure to PM_2.5_ and admission for AMI, MINOCA, and MI-CAD stratified by season**

Top panel: A map of Japan stratified by the 47 prefectures shows seasonal variation of concentration of ambient PM_2.5_ in (a) spring (March–May), (b) summer (June–August), (c) autumn (September–November), and (d) winter (December–February). Bottom panel: This figure shows the odds ratio and 95% confidence interval of the lag effect of the short-term increase of 10 µg/m^3^ in PM_2.5_ stratified by season, indicating the great effect of PM_2.5_ exposure on lag-2 day on admission for AMI, MI-CAD, and MINOCA during spring. Odds ratios and 95% confidence intervals were adjusted for natural cubic splines of temperature (Day 02), and humidity (Day 02) with three knots.

AMI, acute myocardial infarction; PM_2.5_, particulate matter with an aerodynamic diameter ≤2.5 µm; MI-CAD, myocardial infarction with coronary artery disease; MINOCA, myocardial infarction with non-obstructive coronary arteries.

**Figure S4: National-average daily mobility patterns in Japan during the COVID-19 pandemic**

The figure shows the national mean of three Google Community Mobility indices, including workplace (blue line), transit stations (green line), and residential (red line), expressed as percentage change from the pre-pandemic baseline (median for the corresponding weekday during 3 January to 6 February 2020). Each series was smoothed with a 7-day moving average to attenuate day-to-day fluctuation. Pale pink shading highlights the spring season of each year (1 March to 31 May). The orange long-dashed vertical line indicates 7 April 2020, when Japan’s first nationwide COVID-19 state of emergency was declared.

**Figure S5: Seasonal variation and monthly trend for PM_2.5_ concentration in Japan**

Left panel: Summary of ambient PM_2.5_ concentration stratified by season and region. Right panel: A line graph shows the monthly change of concentration of ambient PM_2.5_ in East, West, and All Japan.

**Table S1: A Summary of the number of monitoring stations, distance between the hospital and the nearest station, and value of data.**

|  | Stations, n | Distance (km) | | value |
| --- | --- | --- | --- | --- |
|  |  | Mean (SD) | Median [IQR] |  |
| Meteorological parameters |  |  |  |  |
| Ambient temperature | 466 | 29.4 (89.4) | 4.95 [2.09-23.1] | 1hr average |
| Humidity | 405 | 31.0 (90.0) | 5.92 [2.25-28.6] | 1hr average |
| Air pollutants |  |  |  |  |
| Ox, ppb | 1299 | 4.27 (15.4) | 1.84 [1.07-2.89] | 1hr maximum |
| NO_2_, ppb | 1866 | 2.55 (9.22) | 1.47 [0.87-2.39] | 1hr average |
| SO_2_, ppb | 1226 | 3.75 (10.4) | 2.11 [1.15-3.59] | 1hr average |
| PM_2·5_, μg/m^3^ | 1179 | 3.11 (9.41) | 1.85 [1.08-2.99] | 1hr average |

Ox indicates photochemical oxidants; NO_2_, nitrogen dioxide; SO_2_, sulfur dioxide; PM_2.5_, particulate matter with an aerodynamic diameter≤ 2.5µm.

**Table S2: Baseline characteristics of the study population**

|  | AMI  n=270,091 | MI-CAD  n=247,054 (91.5%) | MINOCA  n=23,037 (8.5%) |
| --- | --- | --- | --- |
| Case days, n | 270,091 | 247,054 | 23,037 |
| Control days, n | 916,366 | 838,308 | 78,058 |
| Age, median (IQR) |  | 70 (61-79) | 71 (60-80) |
| ≥20 to <50 years, n (%) | 22,717 (8.4) | 20,389 (8.3) | 2,328 (10.1) |
| ≥50 to <65 | 66,571 (24.7) | 61,297 (24.8) | 5,274 (22.9) |
| ≥65 to <75 | 78,079 (28.9) | 72,037 (29.2) | 6,042 (26.2) |
| ≥75 | 102,713 (38.0) | 93,323 (37.8) | 9,390 (40.8) |
| Males, n (%) | 201,460 (74.6) | 186,474 (75.5) | 14,986 (65.1) |
| Body mass index, kg/m^2^, median (IQR) | 23.6 (21.3-26.0) | 23.6 (21.4-26.1) | 23.0 (20.7-25.6) |
| Body mass index, not recorded, n (%) | 20,147 (7.5) | 17,966 (7.3) | 2,181 (9.5) |
| Smoking, n (%) | 127,557 (47.2) | 118,310 (47.9) | 9,247 (40.1) |
| Smoking, not recorded, n (%) | 33,080 (12.3) | 29,934 (12.1) | 3,146 (13.7) |
| Killip classification, n (%) |  |  |  |
| Killip 1 | 129,906 (48.1) | 120,587 (48.8) | 9,319 (40.5) |
| Killip 2 | 65,140 (24.1) | 61,283 (24.8) | 3,857 (16.7) |
| Killip 3 | 21,127 (7.8) | 19,569 (7.9) | 1,558 (6.8) |
| Killip 4 | 32,438 (12.0) | 30,677 (12.4) | 1,761 (7.6) |
| Unknown | 21,480 (8.0) | 14,938 (6.0) | 6,542 (28.4) |
| Killip ≥3 | 53,565 (19.8) | 50,246 (20.3) | 3,319 (14.4) |
| Cardiac arrest, n (%) | 9,531 (3.5) | 8,981 (3.6) | 550 (2.4) |
| Hypertension, n (%) | 166,652 (61.7) | 155,609 (63.0) | 11,043 (47.9) |
| Dyslipidemia, n (%) | 164,950 (61.1) | 157,109 (63.6) | 7,841 (34.0) |
| Diabetes, n (%) | 83,884 (31.1) | 77,939 (31.6) | 5,945 (25.8) |
| Atrial fibrillation, n (%) | 17,866 (6.6) | 15,494 (6.3) | 2,372 (10.3) |
| Chronic pulmonary disease, n (%) | 7,406 (2.7) | 6,605 (2.7) | 801 (3.5) |
| Peripheral vascular disease, n (%) | 8,528 (3.2) | 7,994 (3.2) | 534 (2.3) |
| Cerebrovascular disease, n (%) | 12,426 (4.6) | 11,051 (4.5) | 1,375 (6.0) |
| Liver disease, n (%) | 3,332 (1.2) | 2,901 (1.2) | 431 (1.9) |
| Renal disease, n (%) | 16,050 (5.9) | 14,154 (5.7) | 1,896 (8.2) |
| Malignancy, n (%) | 8,706 (3.2) | 7,641 (3.1) | 1,065 (4.6) |
| Low ADL status on admission, n (%) | 193,500 (71.8) | 178,806 (72.5) | 14,694 (64.0) |
| ADL status on admission, not recorded, n (%) | 515 (0.2) | 451 (0.2) | 64 (0.3) |

ADL, activities of daily living; AMI, acute myocardial infarction; IQR, interquartile range; MI-CAD, myocardial infarction with coronary artery disease; MINOCA, myocardial infarction with non-obstructive coronary arteries.

**Table S3: Sensitivity analysis using alternative mobility index**

|  | |  | AMI | | MI-CAD | | MINOCA | |
| --- | --- | --- | --- | --- | --- | --- | --- | --- |
|  | | | OR (95% CI) | P for  interaction | OR (95% CI) | P for  interaction | OR (95% CI) | P for  interaction |
| Meteorological variables + workplace | | |  |  |  |  |  |  |
|  | Pre-pandemic | | 1.101 (1.020-1.189) | 0.073 | 1.085 (1.000-1.176) | 0.248 | 1.298 (1.002-1.683) | 0.018 |
|  | Post-pandemic | | 1.088 (1.014-1.166) |  | 1.076 (0.999-1.158) |  | 1.226 (0.970-1.551) |  |
| Meteorological variables + workplace + transit stations | | |  |  |  |  |  |  |
|  | Pre-pandemic | | 1.101 (1.020-1.189) | 0.073 | 1.085 (1.000-1.176) | 0.246 | 1.299 (1.002-1.683) | 0.020 |
|  | Post-pandemic | | 1.088 (1.014-1.166) |  | 1.075 (0.999-1.158) |  | 1.227 (0.970-1.552) |  |
| Meteorological variables + workplace + residential | | |  |  |  |  |  |  |
|  | Pre-pandemic | | 1.101 (1.020-1.190) | 0.076 | 1.085 (1.000-1.176) | 0.250 | 1.303 (1.005-1.689) | 0.019 |
|  | Post-pandemic | | 1.088 (1.014-1.167) |  | 1.076 (0.999-1.158) |  | 1.231 (0.974-1.557) |  |

ORs and 95% CIs of short-term exposure to PM_2.5_ were adjusted for natural cubic splines of temperature (Day 02), humidity (Day 02), and annual average levels of PM_2.5_ with three knots in the single-pollutant model.

AMI: acute myocardial infarction; CI: confidence interval; MI-CAD: myocardial infarction with coronary artery disease; MINOCA: myocardial infarction with nonobstructive coronary arteries; OR: odds ratio.
